# Genomic findings in patients with clinical suspicion of 22q11.2 deletion syndrome

**DOI:** 10.1007/s13353-016-0366-1

**Published:** 2016-09-14

**Authors:** Magdalena Koczkowska, Jolanta Wierzba, Robert Śmigiel, Maria Sąsiadek, Magdalena Cabała, Ryszard Ślężak, Mariola Iliszko, Iwona Kardaś, Janusz Limon, Beata S. Lipska-Ziętkiewicz

**Affiliations:** 1 Department of Biology and Genetics, Medical University of Gdansk, 1 Debinki Street, 80-211 Gdansk, Poland; 2 General Nursery, Medical University of Gdansk, 7 Debinki Street, 80-211 Gdansk, Poland; 3 Department of Social Pediatrics, Wroclaw Medical University, 5 Bartla Street, 51-618 Wroclaw, Poland; 4 Department of Genetics, Wroclaw Medical University, 1 Marcinkowskiego Street, 50-368 Wroclaw, Poland

**Keywords:** 22q11.2 deletion syndrome, Array comparative genomic hybridization, 17q21.31 microdeletion syndrome, *NF1* microduplication syndrome, chromosome 6p25.3p25.2 deletion, *NAALADL2*

## Abstract

Chromosome 22q11.2 deletion syndrome, one of the most common human genomic syndromes, has highly heterogeneous clinical presentation. Patients usually harbor a 1.5 to 3 Mb hemizygous deletion at chromosome 22q11.2, resulting in pathognomic *TBX1, CRKL* and/or *MAPK1* haploinsufficiency. However, there are some individuals with clinical features resembling the syndrome who are eventually diagnosed with genomic disorders affecting other chromosomal regions. The objective of this study was to evaluate the additive value of high-resolution array-CGH testing in the cohort of 41 patients with clinical features of 22q11.2 deletion syndrome and negative results of standard cytogenetic diagnostic testing (karyotype and FISH for 22q11.2 *locus*). Array-CGH analysis revealed no aberrations at chromosomes 22 or 10 allegedly related to the syndrome. Five (12.2 %) patients were found to have other genomic imbalances, namely 17q21.31 microdeletion syndrome (MIM#610443), 1p36 deletion syndrome (MIM#607872), *NF1* microduplication syndrome (MIM#613675), chromosome 6pter-p24 deletion syndrome (MIM#612582) and a novel interstitial deletion at 3q26.31 of 0.65 Mb encompassing a dosage-dependent gene *NAALADL2*. Our study demonstrates that the implementation of array-CGH into the panel of classic diagnostic procedures adds significantly to their efficacy. It allows for detection of constitutional genomic imbalances in 12 % of subjects with negative result of karyotype and FISH targeted for 22q11.2 region. Moreover, if used as first-tier genetic test, the method would provide immediate diagnosis in ∼40 % phenotypic 22q11.2 deletion subjects.

**Electronic supplementary material:**

The online version of this article (doi:10.1007/s13353-016-0366-1) contains supplementary material, which is available to authorized users.

## Introduction

Chromosomal rearrangements of 22q11.2 are implicated in several genomic disorders, microdeletion 22q11.2 being the most common recurrent syndrome with a prevalence of ∼1:4000–1:6000 live births (Grati et al. 2015). The phenotype of patients with the deletion at 22q11.2 chromosome encompasses a number of clinical entities, including DiGeorge syndrome (DGS; MIM#188400) and velocardiofacial syndrome (VCFS; MIM#192430). The cardinal features comprise conotruncal heart defects, characteristic dysmorphic facies (unusually shaped ears, long nose with broad bridge, micrognathia and upslanting, short palpebral fissures) and velophryngeal insufficiency or cleft palate. Individuals with the more severe phenotype present T-cell immunodeficiency and persistent hypocalcaemia (Tobias et al. 1999; Bassett et al. 2011). Moreover, the presence of some features is determined by the age of individual, for instance psychiatric disorders are more commonly observed in adults (Schneider et al. 2014).

In 90 % of patients with the 22q11.2 deletion, a common ∼3 Mb deletion has been found, whereas 7 % of cases share a smaller, nested ∼1.5 Mb recurrent deletion. Among the remaining individuals atypical deletions or chromosomal translocations involving 22q11.2 have been reported (Schwinger et al. 2010). So far, haploinsufficiency of three genes (*TBX1, CRKL* and *MAPK1*) on 22q11.2 has been reported strongly associated with the syndrome (Schwinger et al. 2010; Breckpot et al. 2012; Rump et al. 2014; Racedo et al. 2015).

Due to the wide variability in the clinical presentation, the diagnosis based on phenotype evaluation is challenging. Even though the state-of-art diagnostic procedure for patients with the clinical suspicion of the 22q11.2 deletion syndrome is the targeted FISH testing, at present patients are usually diagnosed by indirect whole-genome studies, array-CGH being the case in point. Indeed, array-CGH has been recommended as the first-tier diagnostic test for patients with multiple congenital anomalies, developmental delay/intellectual disability and/or autism spectrum disorders; the indications fulfilled also with respect to 22q11.2 deletion syndrome (Henderson et al. 2014).

The aim of the current study was to estimate the efficacy of implementation of the array-CGH screening into the panel of classic diagnostic procedures in subjects resembling the 22q11.2 deletion phenotype. Accordingly, we have applied array-CGH technique to evaluate the incidence and to characterize genomic disorders in the group of patients with clinical features of 22q11.2 deletion syndrome and a normal karyotype and negative result of FISH for 22q11.2 *locus*.

## Clinical report

### Enrollment procedure

Patients from two clinical genetics outpatient clinics (at Gdansk and Wroclaw Medical Universities) were suspected of 22q11.2 deletion syndrome in view of the Tobias criteria (see below) (Tobias et al. 1999). In the years 2004–2014 the confirmation rate of the syndrome established through FISH studies performed using the commercially available D22S75/N25 probe (Cytocell) was 15.8 %. In addition, five patients (11.8 %) were diagnosed with a chromosomal aberration in view of matched karyotype studies. The findings included two cases of a familial translocation (4;11); one case of an unbalanced (X;6) translocation, one case of an unbalanced (14;18) translocation and one case of a large interstitial deletion at chromosome 6q. The remaining patients with the phenotype of 22q11.2 deletion syndrome and with normal results of the standard diagnostic testing (karyotype and targeted FISH) were contacted retrospectively and after obtaining informed consent eventually enrolled in the study.

### Cohort description

The patients were evaluated by two clinical geneticists in view of the Tobias criteria (Tobias et al. 1999). Detailed clinical description of the patients is presented in Supplementary data 1. In brief, 20 (49 %) and 21 (51 %) out of 41 patients met one of the A criteria and at least two of the B criteria, respectively. None of the individuals were diagnosed based on the C criteria. Cardiac defects were detected in 90 % of patients (*n* = 37), including a conotruncal anomaly and Fallot’s tetralogy in 51 % (19/37) and 16 % (6/37), respectively. Characteristic facial features associated with 22q11.2 deletion syndrome were described in 33 patients (80 %); the most frequent being micro(retro)gnathia (58 %), unusually shaped ears (55 %), long nose with broad bridge (52 %) and short palpebral fissures (30 %). Learning difficulties and/or developmental delay were reported in 24 individuals (59 %), whereas 17 % and 5 % of cases were diagnosed with cleft palate and velopharyngeal insufficiency, respectively. Moreover, a total of 16 patients (39 %) had swallowing difficulties. Other anomalies, such as short stature and renal abnormalities were found in 17 % and 15 % of patients, respectively. Only 5 % (2/38) of patients had primary immunodeficiency while none presented with persistent hypocalcaemia.

### Methods

Array-CGH was performed at resolution of 25 kb using Human CGH 3x720K Whole-Genome Tilling Array (Roche, Basel, Switzerland) following the instructions provided by manufacturer with modifications as previously described (Ronowicz et al. 2012). All identified genomic imbalances were verified in the general access databases of genomic variants (DGV, ISCA and Decipher; last accessed December 2015). In selected cases, the *de novo*/familial origin of the aberration was established using qPCR technique. The study was approved by the Research Ethics Committee of Medical University of Gdansk.

### Results

No genomic aberrations at chromosomes 10 and 22 purportedly related to the syndrome were detected. Five patients (12.2 %) were found to have a submicroscopic genomic imbalances of the mean size of 1.5 Mb (Table 1). Patient 29 harbored a 2.1 interstitial deletion of chromosome 1p36.33p36.32, encompassing a total number of 82 genes, including the *GABRD* and *SKI* genes, the region typical for the 1p36 deletion syndrome (MIM#607872). In the individual 22 array-CGH analysis revealed a 2.7 Mb terminal deletion of chromosome 6p25.3p25.2, the minimal region of chromosome 6pter-p24 deletion syndrome (MIM#612582), covering a total number of 18 genes, among which *FOXC1* is proposed as the candidate gene correlating with the reported phenotype. Also, two individuals had interstitial aberrations at chromosome 17. Patient 15 presented with an 1.5 Mb duplication at 17q11.2, whereas a small deletion of 0.5 Mb at 17q21.31 was found in patient 18. The findings correspond with clinical diagnosis of *NF1* microduplication syndrome (MIM#613675) and Koolen–de Vries syndrome (MIM#610443) respectively. A comparison of overlapping and distinct clinical features between the detected genomic disorders and the phenotype associated with 22q11.2 deletion is presented in Table 1.

**Table 1:** Genomic imbalances detected using array-CGH technique in a series of 41 patients presenting 22q11.2 deletion phenotype and negative results of karyotype and FISH for 22q11.2 *locus* studies

Case ID	Results	Size [Mb]	Known recurrent genomic disorder	Key genes for the observed phenotype	Overlapping clinical features between the detected genomic disorder and 22q11.2 deletion observed in the patient	Distinct clinical features between the detected genomic disorder and 22q11.2 deletion observed in the patient
29	arr[hg18] 1p36.33p36.32(689,001-2,833,131)x1	2.1	1p36 deletion syndrome (MIM#607872)	*GABRD, SKI*	developmental delay, feeding (swallowing) difficulty in infancy, congenital heart defect (valvular defect), epilepsy	dysmorphism: midface hypoplasia, pointed chin, fifth finger clinodactyly brachydactyly; severe ID; does not speak at 15 y.o;* temper tantrums*
41	arr[hg18] 3q26.31(176,570,887-177,223,154)x1	0.7	-	*NAALADL2*	*n/a*	*n/a*
22	arr[hg18] 6p25.3p25.2(0–2,740,688)x1	2.7	Chromosome 6pter-p24 deletion syndrome (MIM#612582)	*FOXC1*	short stature, congenital heart defect (valvular defect), swallowing difficulties - high-arched palate; vesicoureteral reflux, depressive syndrome	dysmorphism: midface hypoplasia, hypertelorism, down-slanting palpebral fissures, flat nasal bridge
15	arr[hg18] 17q11.2(25,927,664-27,350,462)x3	1.5	*NF1* microduplication syndrome (MIM#613675)	*NF1, RNF135*	dysmrophism: tubular nose, abnormal ears, micrognathia; cleft palate	*–*
18	arr[hg18] 17q21.31(41,071,028-41,569,975)x1	0.5	Koolen-de Vries syndrome (MIM#610443)	*KANSL1*	developmental delay, congenital heart defect, epilepsy, swallowing difficulties, vesicoureteral reflux, amblyopia	dysmorphism: long face, epicanthal folds, pear-shaped nose, broad chin, hip dislocation

Legend: *MIM* Mendelian Inheritance in Man Database reference number, *n/a* not applicable

Finally, a unique loss at chromosome 3q26.31 of 650 kb encompassing the entire *NAALADL2* gene sequence, was identified in patient 41. qPCR analysis revealed the same deletion in two siblings of the patient (a sister with bilateral cleft lip and palate and a brother with congenital heart defect) and their apparently unaffected mother (for details see Supplementary data 2).

## Discussion

Nowadays, the gold standard diagnostic procedure for 22q11.2 deletion syndrome is by conventional cytogenetic technique and targeted FISH analysis (Schwinger et al. 2010). Several studies have demonstrated that according to this approach the detection rate varies considerably (4–96 %) (Yagi et al. 2003; Smigiel et al. 2007; Brunet et al. 2009; Fernandez et al. 2008; Wozniak et al. 2010). However, a more practical approach is to perform a whole-genome analysis, as it not only allows one to identify the syndrome but at the same time may detect any other genomic syndrome presenting overlapping clinical features. To the best of our knowledge, our work is the first attempt to evaluate application of aCGH technique in the cohort of patients presenting phenotype resembling the 22q11.2 deletion syndrome.

To date, over 180 clinical features have been associated with the 22q11.2 deletion syndrome. Clinical diagnosis of 22q11.2 deletion syndrome remains a challenging task, because no single pathognomonic finding exists for the syndrome. Even though it has been postulated that the two major striking phenotypic features are: immunodeficiency and hypocalcaemia (Bassett et al. 2011), that was not shown in the large series of patients. Similar to the current series, in the recent report of almost 750 cases diagnosed in France over last 18 years the most frequent reasons for referral of postnatally diagnosed cases were a congenital heart defect (49 %), facial dysmorphism (50 %) and developmental delay (41 %). Hypocalceamia was present in 15 % only, while immunodeficiency was not among referral causes; instead recurrent infections were reported in 8 % and thymus agenesis in 7 %. Actually, 25 % of the patients were referred because of a single feature; of these, half had a congenital heart defect only (Poirsier et al. 2015).

**Fig. 1:**
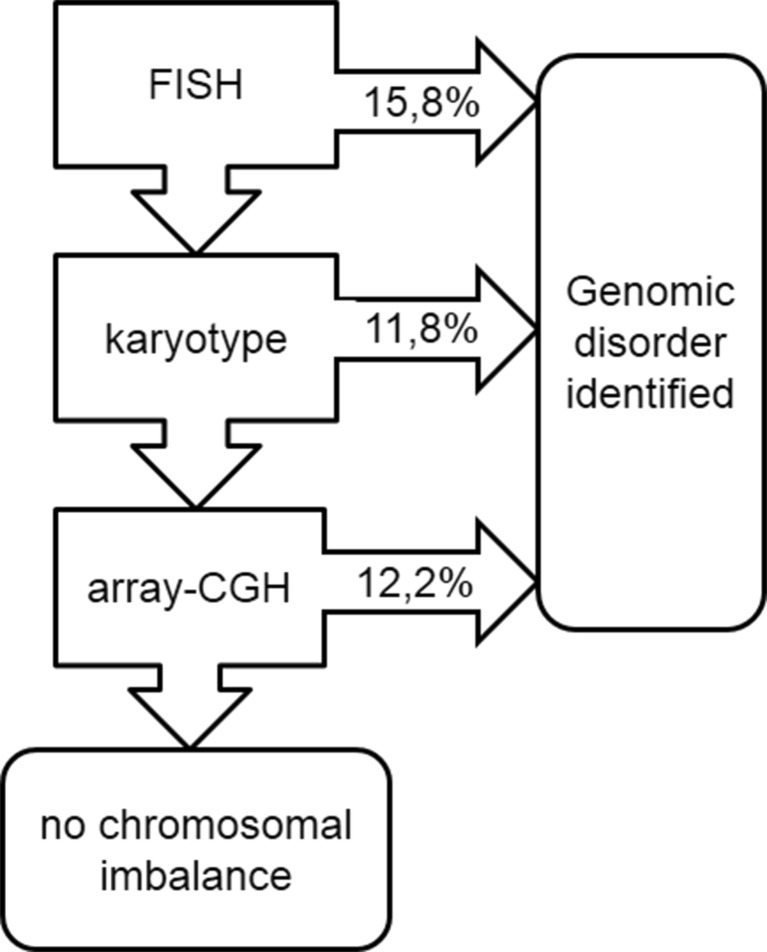
The flow-chart showing subsequent detection rates of genomic disorders in patients with the clinical features of 22q11.2 deletion syndrome using various testing approaches (targeted FISH followed by conventional karyotyping, followed by array-CGH)

In the last ten years, the clinical diagnosis of the syndrome has been confirmed by FISH in 16 % of the patients referred to our Outpatient Genetic Clinics (Fig. 1). Concurrent conventional cytogenetic analysis allowed us to identify genomic imbalances as the underlying cause of the observed phenotype in additional 12 % of cases.

In this study, 41 patients with clinical signs of 22q11.2 deletion syndrome and with negative standard diagnostic analysis were further evaluated using array-CGH. Five individuals (12.2 %) were diagnosed to harbor pathogenic genomic imbalances, including four known recurrent genomic disorders (Table 1). Our findings add on to the clinical spectrum of these syndromes and show that their clinical manifestation might mimic the presentation of the 22q11.2 deletion syndrome. Nonetheless, these patients lacked cardinal clinical signs, such as conotruncal heart defects or family history of 22q11.2 deletion syndrome; also their dysmorphic features were distinct from the 22q11.2 deletion phenotype in three of four individuals. Based on the results of array analysis reverse phenotyping was performed revealing incomplete phenotype in all of them (Table 1). The patient (#22) with a 2.7 Mb deletion at chromosome 6p25 did not present anterior eye chamber abnormalities (including Axenfeld-Rieger syndrome type 3 in particular) that are considered pathognomonic for the syndrome. Also, the boy (#15) with a 1.5 duplication at 17q11.2 encompassing *NF1* gene did not present developmental delay or seizures allegedly reported in patients with such duplication. The girl (#18) with a 0.5 Mb deletion at 17q21.31 presented mild dysmorphic features and lacked blepharophimosis, ptosis, or abnormal hair pigmentation and texture usually observed in children with Koolen-de Vries syndrome. The girl (#29) with a 2.1 Mb deletion at chromosome 1p36 did not have structural abnormalities of the brain, vision or hearing impairment or other malformations usually present in children with the syndrome.

In addition to known recurrent syndromes, we identified a unique deletion at 3q26.31 of 0.65 Mb size. The deletion encompasses only one dosage-dependent gene, *NAALADL2* (MIM*608806), that is thought to be associated with the congenital defects, dysmorphic features and/or development delay (Millson et al. 2012). Family study (Supplementary data 2) revealed the variant to be present in two affected siblings of the patient and an apparently healthy mother indicating reduced penetrance and variable expressivity.

In the current study no submicroscopic chromosomal rearrangements at chromosome 22 or 10 allegedly related to the syndrome were identified. The losses at DGS2 *locus* located at 10p13p14 chromosome have an estimated frequency of one in 200,000 live births (Daw et al. 1996; Lindstrand et al. 2010). The DGS2 clinical presentation is thought to be more severe than the regular 22q11.2 deletion syndrome with severe mental retardation in addition to immune deficiency and heart defects. Due to its rarity, and in line with the fact, that only a small fraction of the subjects studied in the current work presented with such a severe phenotype, our study might have disregarded such cases. Otherwise, our results are in line with the study of Bartsch et al. (1999), suggesting no evidence for genomic deletions at putative DGS2 locus on 10p in phenotypic 22q11.2 deletion patients.

The recommended optimal resolution for array-CGH used in routine diagnostics, that does not increase greatly the number of uncertain significance variants, is 200 kb (Vermeesch et al. 2012). In the current study we have used high-resolution array-CGH at 25 kb which did not improve diagnostic yield. It only increased the background noise related to the detected small CNVs of unknown significance (on average 2 per sample), eventually proven to be insignificant to the reported phenotype. Plausibly, the high resolution could be useful in detecting small aberrations, affecting the locus of *TBX1* gene in particular as previously reported by Chen et al. (2014), but no atypical small aberration at 22q11.2 *locus* was observed in the current series of patients.

Taking into account the high variability of phenotype in patients with the clinical suspicion of 22q11.2 deletion syndrome, we recommended to use array-CGH as the first-line genetic test, in place of conventional cytogenetic procedures and targeted FISH. Array-CGH has proven to increase the resolution and provide higher diagnostic yield than classical karyotyping which improves diagnostic capability (Miller et al. 2010; Kaminsky et al. 2013). In the presented study, if array-CGH had been used as the first-tier clinical diagnostic test it could have confirmed the diagnosis of a genomic disorder in ∼40 % of individuals with clinical manifestation of the 22q11.2 deletion syndrome (Fig. 1). The resolution of the technique without any doubt could detect the imbalances present in the patients diagnosed at previous stages of diagnostic procedures (i.e. by FISH or conventional karyotyping). Array-CGH appears not only as less time-consuming but also as the cost-effective technique. In the remaining 60 % of patients, the screening for detection of unknown gene mutations by whole-exome sequencing (WES) should be considered. In line with the recommendations of the American College of Medical Genetics and Genomics (2012) and the European Society of Human Genetics (2013) WES analysis should be considered in the clinical evaluation of individuals with suspected genetic disorders only under certain circumstances. Interpretation of an enormous amount of raw data and the risk of identification of incidental (secondary) findings are the main limiting factors (ACMG 2012; van El et al. 2013).

In conclusion, our study confirms that array-CGH screening of individuals with clinical suspicion of 22q11.2 and the negative result of standard genetic testing increases the capacity to detect submicroscopic chromosomal aberrations and therefore allows one to make the proper diagnosis in a significant fraction of patients. Accordingly, we recommend array-CGH testing as the first choice test in this particular clinical setting.

## Electronic supplementary material

Below is the link to the electronic supplementary material.

ESM 1 (PDF 312 kb)
