# Supplementary material for: Genomic findings in patients with clinical suspicion of 22q11.2 deletion syndrome
Source: J Appl Genet. 2016 Sep 14;58(1):93–8. doi: 10.1007/s13353-016-0366-1 (PMC5243916; doi:10.1007/s13353-016-0366-1)
Supplement: Supplementary file 1 — (PDF 312 kb) [file 13353_2016_366_MOESM1_ESM.pdf]

Magdalena Koczkowska, Jolanta Wierzba, Robert Smigiel, Maria Sasiadek, Magdalena Cabala, Ryszard Ślęzak, Mariola Iliszko, Iwona Kardas, Janusz Limon, Beata S. Lipska-Zietkiewicz.

Supplementary Data 1.
Incidence of clinical features typical for deletion 22q11.2 syndrome in 41 patients enrolled in the study.

F – Female, M – Male, R – Right, L – Left, B – Bilateral, nd – no data. Blank boxes indicate absence of the feature.

		Congenital heart defect			Craniofacial anomalies									Other anomalies								
Patient	Gender	Heart defect, icluding:	* Conotruncal anomaly	* Fallot's tetralogy	Micro(retro)gnathia	Long nose with broad bridge	Unusually shaped ears	Long slender fingers and hands	Short palpebral fissures	Facial asymmetry	Cleft palate	Velopharyngeal insufficiency	Swallowing difficulty	Learning difficulties / developemtal delay	Epilepsy	Psychiatric disorder	Immunodefficiency / thymic hypoplasia	Hypocalcaemia	Renal abnormalities / Potter sequence	Short stature	Family history
1	M	+			+		+		+					+	+					+	
2	M	+			+			+					+	+		+					
3	M				+		+							+							
4	M	+	+																		
5	M	+	+	+								+	+						R		
6	M	+				+	+							+							
7	F	+	+	+		+		+					+	+						+	
8	F	+								+											
9	M	+	+			+	+							+							
10	F	+	+	+	+	+								+							
11	F	+	+			+															
12	M	+			+			+					+	+		+					
13	F	+			+								+	+						+	
14	F	+	+		+		+							+							
15	M				+	+	+		+		+										
16	M	+	+			+	+				+			+						+	
17	F	+	+	+																	
18	F	+											+	+	+				B		

**19**	F	+	+			+	+							+							
**20**	F	+				+		+		+		+									
**21**	M	+	+	+		+	+		+	+			+								
**22**	F	+	+					+					+			+			+		
**23**	M	+	+		+								+	+						+	
**24**	M	+	+											+			+				
**25**	F	+			+			+			+		+	+							
**26**	M	+			+		+						+								
**27**	M	+				+	+		+	+				+							
**28**	F												+	+	+						
**29**	F	+												+	+	+					
**30**	F	+	+		+		+				+		+	+			nd				
**31**	M	+	+	+		+															
**32**	M	+			+		+							+							
**33**	M	+	+			+	+										+				
**34**	M	+			+		+		+		+		+	+					B	+	
**35**	F	+	+		+	+	+		+	+	+			+							
**36**	M	+			+	+	+		+	+									L		
**37**	M	+			+	+	+		+	+										+	
**38**	F	+			+				+				+								
**39**	M	+	+														nd	nd	+		
**40**	F	+				+					+		+				nd	nd			
**41**	F				+				+					+							+

Supplementary Data 2.
Co-segregation of the unique 0.65Mb deletion at chromosome 3q26 in Family #41.

A. Family pedigree.

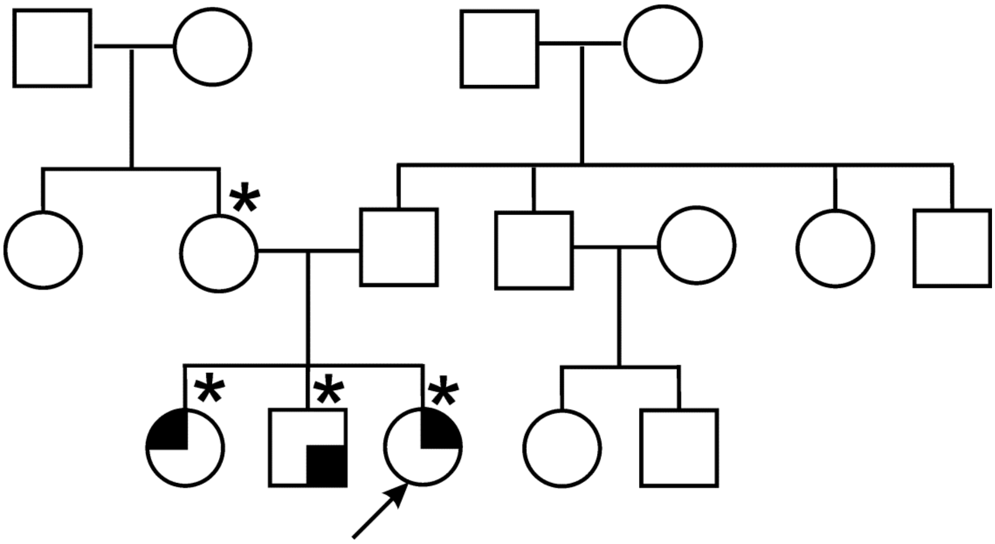

Legend:
* - carriers of arr [hg18] 3q26.31(176,570,887-177,223,154)x1

B. Clinical data.

Affected family members:

Older sister (aged 17) – IUGR, bilateral cleft lip and cleft palate, short stature, congenital hypothyroidism, strabismus (exotropia), normal motor and psychosocial development, recurrent infections; dysmorphic features: long face, deep-set eyes, long nose with broad bridge, short philtrum, full triangle-shaped lips, long slender fingers of hands and feet.

Older brother (aged 16) – IUGR, hemodynamically significant congenital heart defect (VSD+PDA; surgical correction in infancy); normal motor and psychosocial development, dysmorphic features: long face, hypotelorism, long nose with broad bridge, short philtrum, small lips, long slender fingers of hands and feet.

Patient #41 (aged 8) – IUGR, no congenital defects, normal motor development, severe intellectual disability, autism spectrum disorder, dysmorphic features: long face, short palpebral fissures, long nose with broad bridge, short philtrum, micrognathia, long slender fingers of hands and feet.

Mother (aged 39) – no perinatal data, no congenital defects, normal motor and psychosocial development, dysmorphic features: long face, hypotelorism, long nose with broad bridge, short philtrum.

Ethnic background: The family is Kashubian, i.e. comes from a small inbred population of Pomerania region of Poland. Even though the parents negate being related, knowledge of the history of the region suggest relatively high possibility of consanguinity.
